# 3D QSAR Studies, Pharmacophore Modeling and Virtual Screening on a Series of Steroidal Aromatase Inhibitors

**DOI:** 10.3390/ijms151120927

**Published:** 2014-11-14

**Authors:** Huiding Xie, Kaixiong Qiu, Xiaoguang Xie

**Affiliations:** 1 Department of Chemistry, Yunnan University, Kunming 650091, Yunnan, China; 2 Department of Chemistry, School of Pharmaceutical Science & Yunnan Key Laboratory of Pharmacology for Natural Products, Kunming Medical University, Kunming 650500, Yunnan, China; E-Mail: chenneyao16@hotmail.com

**Keywords:** steroidal aromatase inhibitors, 3D QSAR, CoMFA, CoMSIA, pharmacophore, virtual screening

## Abstract

Aromatase inhibitors are the most important targets in treatment of estrogen-dependent cancers. In order to search for potent steroidal aromatase inhibitors (SAIs) with lower side effects and overcome cellular resistance, comparative molecular field analysis (CoMFA) and comparative molecular similarity indices analysis (CoMSIA) were performed on a series of SAIs to build 3D QSAR models. The reliable and predictive CoMFA and CoMSIA models were obtained with statistical results (CoMFA: *q*^2^ = 0.636, *r*^2^_ncv_ = 0.988, *r*^2^_pred_ = 0.658; CoMSIA: *q*^2^ = 0.843, *r*^2^_ncv_ = 0.989, *r*^2^_pred_ = 0.601). This 3D QSAR approach provides significant insights that can be used to develop novel and potent SAIs. In addition, Genetic algorithm with linear assignment of hypermolecular alignment of database (GALAHAD) was used to derive 3D pharmacophore models. The selected pharmacophore model contains two acceptor atoms and four hydrophobic centers, which was used as a 3D query for virtual screening against NCI2000 database. Six hit compounds were obtained and their biological activities were further predicted by the CoMFA and CoMSIA models, which are expected to design potent and novel SAIs.

## 1. Introduction

Aromatase is a cytochrome P-450 dependent enzyme that catalyzes the aromatization of androgens to estrogens. Aromatase inhibitors (AIs) reduce the synthesis of estrogens and offer a therapeutic alternative for the treatment of estrogen-dependent cancers, such as breast cancer [1,2,3]. There are two classes of AIs, steroidal and non-steroidal compounds, which cause potent estrogen suppression [4]. The non-steroidal aromatase inhibitors (NSAIs) are mostly azole type compounds, such as the clinically used anastrozole and letrozole, which compete with the substrate for binding to the enzyme active site [5]. Among steroidal aromatase inhibitors (SAIs), formestane was widely used during the early 1990s, but it is not used nowadays because of the need to administer it by intramuscular injection. Therefore, the orally active exemestane is the main steroidal inhibitor [6]. These SAIs mimic the natural substrate androstenedione and are converted by the enzyme to reactive intermediates, which bind irreversibly to the enzyme active site, resulting in inactivation of aromatase [7]. Despite the success of the third-generation NSAIs (anastrazole and letrozole) and SAIs (exemestane), they still have some major side effects, such as increase of bone loss, joint pain, and heart problems [8]. In addition, after some years of usage they can develop cellular resistance. For these reasons, it is important to search for other potent and specific molecules with lower side effects and which can overcome the resistance phenomena.

Quantitative structure-activity relationship (QSAR) methods have been successfully employed to assist the design of new small molecule drug candidates [9,10,11,12,13,14,15,16]. Comparative molecular field analysis (CoMFA) and comparative molecular similarity indices analysis (CoMSIA) are two of the most widely used three-dimensional quantitative structure-activity relationship (3D QSAR) methodologies. CoMFA calculates the energies of steric and electrostatic interactions between the compound and the probe atom at various intersections of a regular 3D lattice according to Lennard-Jones and Coulomb potentials. The resulting energies derived from these two potential functions can be contoured to offer a quantitative spatial description of the molecular properties [17]. CoMSIA introduces the Gaussian function for the distance dependence between the molecular atoms and the probe atom in order to avoid some inherent deficiencies arising from the Lennard-Jones and Coulomb potential functional forms. CoMSIA is applied to gain an insight into how steric fields, electrostatic fields, hydrophobic fields, hydrogen bond donor (HBD) and hydrogen bond acceptor (HBA) influence the activity of inhibitors [18].

Pharmacophore modeling can provide valuable insight into ligand-receptor interactions. Pharmacophore searches are the best option to find a range of chemical structures with viable features. A pharmacophore model can be considered as the ensemble of steric and electrostatic features of different compounds, which are necessary to ensure optimal supramolecular interactions with a specific biological target structure and to trigger or to block its biological response. Thus, pharmacophore modeling is the method of choice for the first round of compound selection. This ability of a pharmacophore model is used to find new classes of inhibitors when one class is known. This is known as “scaffold hopping” [19,20,21].

A series of SAIs, shown in Table 1, have been reported in the recent literatures [22,23,24,25,26,27]. To understand the structural requirements for inhibitory activity and design more potent agents, 3D QSAR studies were performed for the fist time for these SAIs using CoMFA and CoMSIA. In addition, 3D pharmacophore models were created and the selected best model was used as a 3D query for virtual screening against NCI2000 database. The biological activities of hit compounds were further predicted by using CoMFA and CoMSIA models.

**Table 1:** Chemical structures and bioactivity values of the steroidal aromatase inhibitors in current study.

Compound	General Structure	Substituents	IC_50_ (µM)	pIC_50_
1 ^b^	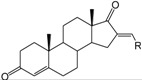	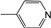	5.2	5.284
2 ^a^		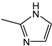	22.7	4.644
3	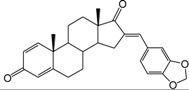		6.4	5.194
4	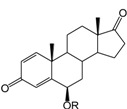	–CH_3_	5.2	5.284
5 ^a^		–CH_2_CH_3_	18.1	4.742
6		–CH_2_C≡CCH_3_	0.1123	6.950
7 ^b^		–CH_2_C≡CCH_2_CH_3_	0.0118	7.928
8 ^a^		–CH_2_C≡C(CH_2_)_2_CH_3_	0.083	7.081
9		–CH_2_C≡C(CH_2_)_3_CH_3_	0.1811	6.742
10		–CH_2_C≡C(CH_2_)_6_CH_3_	2.18	5.662
11		–CH_2_C≡CCH_2_OH	0.02	7.699
12 ^b^ (Exemestane)	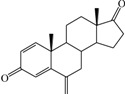		0.0501	7.300
13 ^b^ (Formestane)	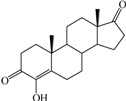		0.0486	7.313
14 ^a^	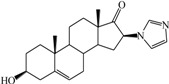		3.30	5.481
15	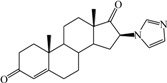		0.18	6.745
16 ^b^	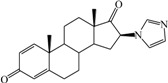		0.16	6.796
17	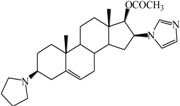		4.90	5.310
18 ^a^	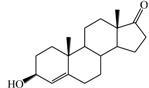		0.183	6.738
19 ^b^	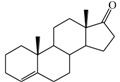		0.135	6.870
20	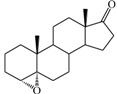		0.970	6.013
21	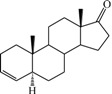		0.225	6.648
22 ^a^	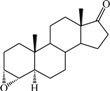		0.145	6.839
23	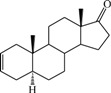		1.733	5.761
24	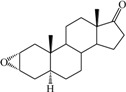		1.150	5.939
25 ^a^	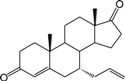		0.59	6.229
26	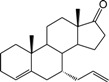		0.75	6.125
27 ^b^	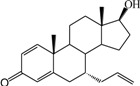		0.45	6.347
28	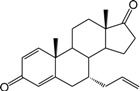		0.47	6.328
29 ^a^	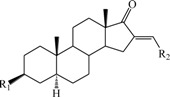	1-OH; 2-Ph	7.27	5.138
30		1-OH; 2-MeOPh(p)	7.13	5.147
31		1-OCOCF3; 2-Ph	7.12	5.148
32		1-OCOCF3; 2-MeOPh(p)	7.00	5.155
33	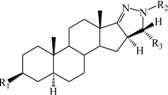	1-OCOCF_3_; 2-COCH_3_; 3-Ph	6.57	5.182
34 ^a^		1-OCOCF_3_; 2-COCH_3_; 3-MeOPh(p)	6.45	5.190
35		1-OCOCF_3_; 2-COCH_2_CH_3_; 3-Ph	6.72	5.173
36		1-OCOCF_3_; 2-COCH_2_CH_3_; 3-MeOPh(p)	6.61	5.180
37		1-OH; 2-H; 3-Ph	6.91	5.161
38		1-OH; 2-H; 3-MeOPh(p)	6.83	5.166
39 ^a^		1-OCOCF_3_; 2-CH_3_; 3-Ph	5.81	5.236
40		1-OCOCF_3_; 2-CH_3_; 3-MeOPh(p)	5.78	5.238
41 ^a^		1-OCOCF_3_; 2-Ph; 3-Ph	5.67	5.246
42		1-OCOCF_3_; 2-Ph; 3-MeOPh(p)	5.45	5.264
43		1-OH; 2-CH_3_; 3-Ph	6.34	5.198
44		1-OH; 2-CH_3_; 3-MeOPh(p)	6.12	5.213
45		1-OH; 2-Ph; 3-Ph	6.01	5.221
46 ^a^		1-OH; 2-Ph; 3-MeOPh(p)	5.92	5.228
47	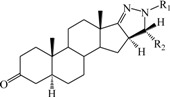	1-H; 2-Ph	5.23	5.281
48		1-H; 2-MeOPh(p)	4.88	5.312
49		1-COCH_3_; 2-Ph	4.91	5.309
50		1-COCH_3_; 2-MeOPh(p)	4.89	5.311
51 ^a^		1-CH_3_; 2-Ph	4.78	5.321
52		1-CH_3_; 2-MeOPh(p)	4.56	5.341
53		1-Ph; 2-Ph	4.27	5.370
54		1-Ph; 2-MeOPh(p)	4.16	5.381
55 ^a^	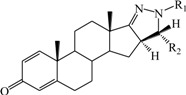	1-COCH_3_; 2-Ph	2.88	5.541
56		1-COCH_3_; 2-MeOPh(p)	2.65	5.577
57		1-H; 2-Ph	3.01	5.521
58		1-H; 2-MeOPh(p)	2.91	5.536
59		1-CH_3_; 2-Ph	2.45	5.611
60 ^a^		1-CH_3_; 2-MeOPh(p)	2.11	5.676
61		1-Ph; 2-Ph	1.98	5.703
62 ^b^		1-Ph; 2-MeOPh(p)	1.82	5.740
63	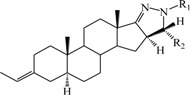	1-CH_3_; 2-Ph	3.51	5.455
64		1-CH_3_; 2-MeOPh(p)	3.40	5.469
65 ^a^		1-Ph; 2-Ph	3.34	5.476
66		1-Ph; 2-MeOPh(p)	3.23	5.491

^a^ Test-set compounds; and ^b^ Compounds used to generate pharmacophore models.

## 2. Results and Discussion

### 2.1. CoMFA and CoMSIA Statistical Results

The statistical parameters of standard CoMFA models constructed with steric and electrostatic fields are given in Table 2. The cross-validated coefficient (*q*^2^/*r*^2^_cv_), non-cross-validated correlation coefficient (*r*^2^_ncv_), standard error estimate (SEE) and *F*-statistic values (*F*) were computed as defined in SYBYL. The cross-validated (leave-one-out) PLS analysis shows a *q*^2^ value of 0.636 with ten components and non-cross-validated PLS analysis results in *r*^2^_ncv_ of 0.988, SEE of 0.094 and *F* value of 309.026, which indicates it is a model with high quality. The corresponding field contributions of steric and electrostatic are 0.671 and 0.329, respectively, which means the steric field gives more contribution to the bioactivity than the electrostatic field does.

**Table 2:** Summary of CoMFA and CoMSIA results.

Components	CoMFA	CoMSIA (SA)
*q*^2^ (*r*^2^_cv_)	0.636	0.843
*r*^2^_ncv_	0.988	0.989
SEE	0.094	0.096
*F* value	309.026	174.304
*r*^2^_pred_	0.658	0.601
No. of compounds	50	50
No. of optimal components	10	17
Contribution		
Steric	0.671	0.677
Electrostatic	0.329	–
Hydrophobic	–	–
H-bond donor	–	–
H-bond acceptor	–	0.323

**Figure 1:**
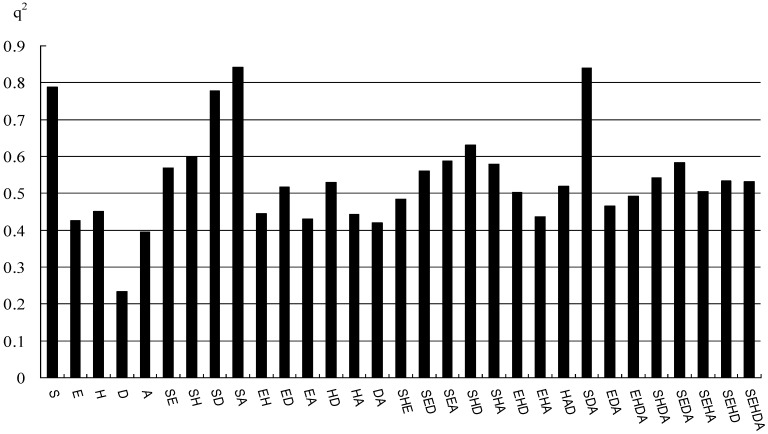
The histogram of 31 possibilities of the CoMSIA field combinations (S = steric, E = electrostatic, H = hydrophobic, D = hydrogen bond donor, A = hydrogen bond acceptor).

Commonly, in CoMSIA, five different similarity fields (steric, electrostatic, hydrophobic, HBD, and HBA) are calculated. However, for CoMSIA models, the model with global descriptors is not the best model in all probability. Some papers [28,29] have discussed whether the five different descriptor fields in CoMSIA are totally independent of each other. The dependencies of the individual fields usually decrease the signal-to-noise ratio in the data [29] and lower the statistical significance of the results. Therefore, in our study, an optimization of 31 possible combinations of five different descriptor fields was evaluated from the values of the *q*^2^, which is shown in Figure 1. The higher the value of *q*^2^ shows, the better the model is. With the highest *q*^2^ of 0.843, CoMSIA model with the combination of steric and HBA fields (SA) was finally chosen as the best model, which indicates that the steric and HBA fields mainly contribute to the binding affinities. This CoMSIA (SA) model was obtained with seventeen optimal components. The analysis results are summarized in Table 2 (*q*^2^ = 0.843, *r*^2^_ncv_ = 0.989, *F* = 174.304, SEE = 0.096). The corresponding field contributions of steric and HBA are 0.677 and 0.323, respectively, which means that the steric field provides more bioactivity contribution than the HBA field does.

### 2.2. Validation of 3D QSAR Models

**Figure 2:**
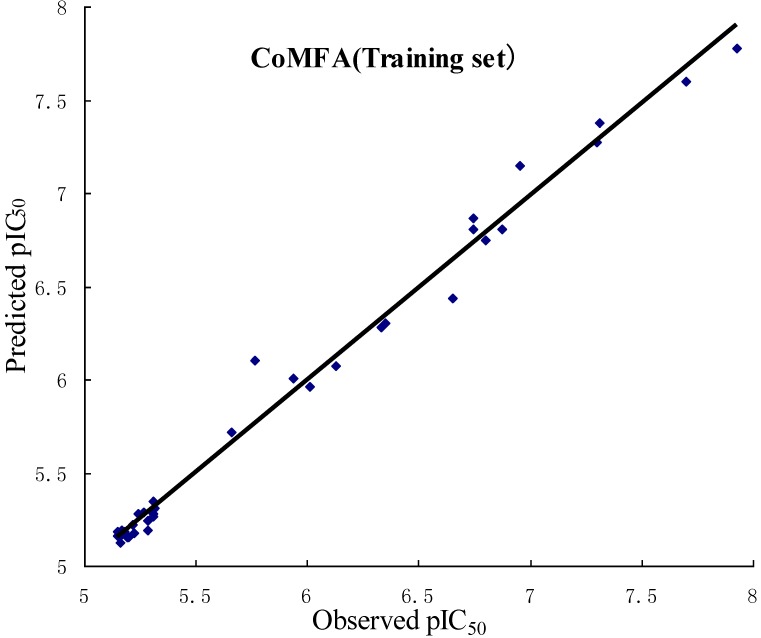

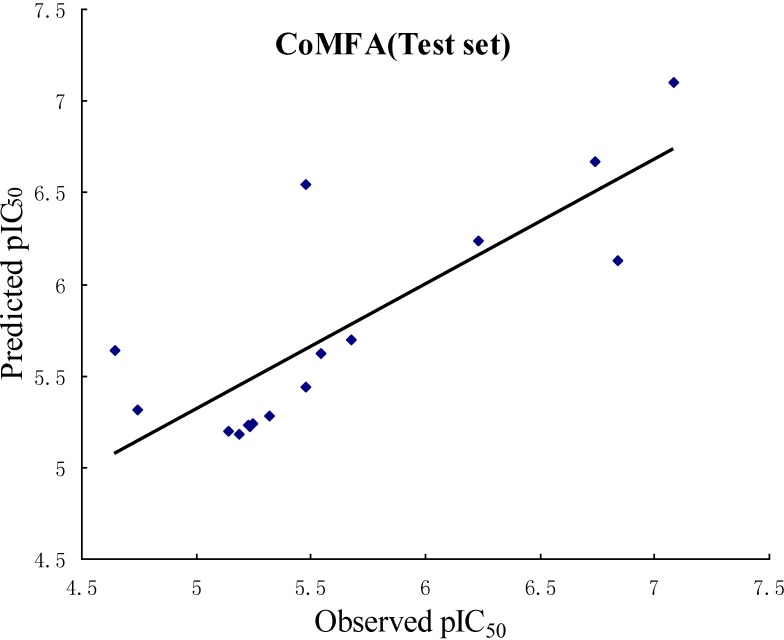
Plots of observed *versus* predicted activities of the training set and test set molecules from CoMFA analysis.

**Figure 3:**
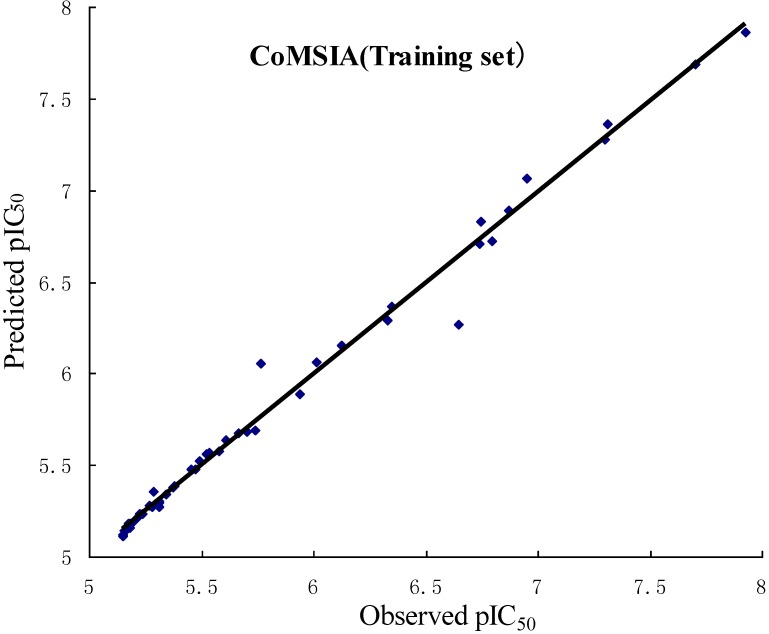

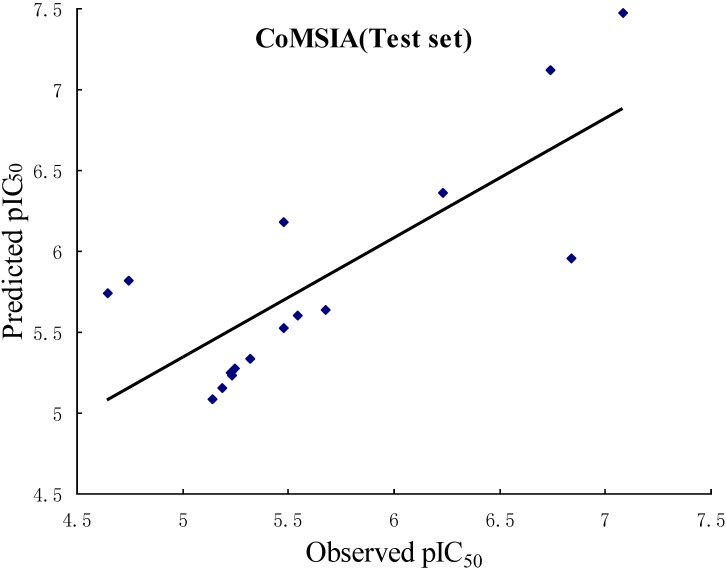
Plots of observed *versus* predicted activities of the training set and test set molecules from CoMSIA analysis.

In order to validate the obtained 3D QSAR models, *r*^2^_pred_ was used to determine the predictive abilities of the CoMFA and CoMSIA models from the 16 compounds (test set), which were not included in the generation of the models. The obtained *r*^2^_pred_ of the test set is 0.658, 0.601 for the CoMFA, CoMSIA model, respectively, which indicates that both models have good predictive ability. The observed and predicted pIC_50_ of the training and test sets by the CoMFA and CoMSIA models are listed in Table 3, and the correlations between the observed and predicted pIC_50_ of training and test sets are depicted in Figure 2 for CoMFA model, Figure 3 for CoMSIA model, respectively.

**Table 3:** Observed and predicted pIC_50_ of the training and test sets using CoMFA and CoMSIA models.

Compound	Observed pIC_50_	CoMFA		CoMSIA	
		Pred.	Res.	Pred.	Res.
1	5.284	5.247	0.037	5.357	−0.073
2 ^a^	4.644	5.642	−0.998	5.744	−1.100
3	5.194	5.152	0.042	5.192	0.002
4	5.284	5.191	0.093	5.278	0.006
5 ^a^	4.742	5.314	−0.572	5.822	−1.080
6	6.950	7.149	−0.199	7.064	−0.114
7	7.928	7.778	0.150	7.867	0.061
8 ^a^	7.081	7.102	−0.021	7.477	−0.396
9	6.742	6.804	−0.062	6.711	0.031
10	5.662	5.722	−0.060	5.678	−0.016
11	7.699	7.598	0.101	7.685	0.014
12	7.300	7.272	0.028	7.281	0.019
13	7.313	7.378	−0.065	7.361	−0.048
14 ^a^	5.481	6.542	−1.061	6.181	−0.700
15	6.745	6.869	−0.124	6.830	−0.085
16	6.796	6.746	0.050	6.726	0.070
17	5.310	5.348	−0.038	5.307	0.003
18 ^a^	6.738	6.668	0.070	7.123	−0.385
19	6.870	6.811	0.059	6.888	−0.018
20	6.013	5.965	0.048	6.062	−0.049
21	6.648	6.436	0.212	6.267	0.381
22 ^a^	6.839	6.132	0.707	5.958	0.881
23	5.761	6.102	−0.341	6.057	−0.296
24	5.939	6.008	−0.069	5.888	0.051
25 ^a^	6.229	6.236	−0.007	6.359	−0.130
26	6.125	6.074	0.051	6.151	−0.026
27	6.347	6.301	0.046	6.365	−0.018
28	6.328	6.282	0.046	6.292	0.036
29 ^a^	5.138	5.202	−0.064	5.084	0.054
30	5.147	5.186	−0.039	5.122	0.025
31	5.148	5.165	−0.017	5.111	0.037
32	5.155	5.154	0.001	5.148	0.007
33	5.182	5.169	0.013	5.184	−0.002
34 ^a^	5.190	5.185	0.005	5.157	0.033
35	5.173	5.184	−0.011	5.186	−0.013
36	5.180	5.187	−0.007	5.160	0.020
37	5.161	5.127	0.034	5.146	0.015
38	5.166	5.194	−0.028	5.153	0.013
39 ^a^	5.236	5.222	0.014	5.233	0.003
40	5.238	5.284	−0.046	5.234	0.004
41 ^a^	5.246	5.237	0.009	5.273	−0.027
42	5.264	5.291	−0.027	5.282	−0.018
43	5.198	5.156	0.042	5.200	−0.002
44	5.213	5.222	−0.009	5.209	0.004
45	5.221	5.177	0.044	5.236	−0.015
46 ^a^	5.228	5.230	−0.002	5.247	−0.019
47	5.281	5.243	0.038	5.270	0.011
48	5.312	5.310	0.002	5.277	0.035
49	5.309	5.265	0.044	5.294	0.015
50	5.311	5.285	0.026	5.271	0.040
51 ^a^	5.321	5.279	0.042	5.337	−0.016
52	5.341	5.341	0.000	5.339	0.002
53	5.370	5.303	0.067	5.377	−0.007
54	5.381	5.356	0.025	5.388	−0.007
55 ^a^	5.541	5.626	−0.085	5.603	−0.062
56	5.577	5.640	−0.063	5.574	0.003
57	5.521	5.594	−0.073	5.565	−0.044
58	5.536	5.656	−0.120	5.569	−0.033
59	5.611	5.633	−0.022	5.638	−0.027
60 ^a^	5.676	5.698	−0.022	5.641	0.035
61	5.703	5.659	0.044	5.682	0.021
62	5.740	5.714	0.026	5.690	0.050
63	5.455	5.408	0.047	5.475	−0.020
64	5.469	5.469	0.000	5.479	−0.010
65 ^a^	5.476	5.442	0.034	5.522	−0.046
66	5.491	5.491	0.000	5.526	−0.035

^a^ Test-set compounds.

### 2.3. CoMFA Contour Maps

**Figure 4:**
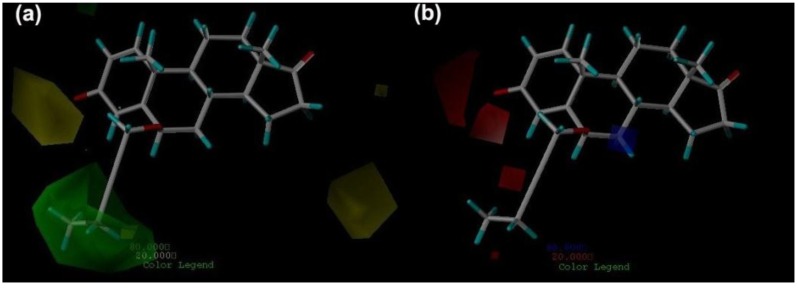
CoMFA contour maps (standard deviation × coefficient) in combination with compound **7**. (**a**) Steric contour maps: Green contours (80% contribution) refer to sterically favored regions, yellow contours (20% contribution) indicate sterically disfavored regions; and (**b**) Electrostatic contour maps: Blue contours (80% contribution) refer to regions where positively charged substituents are favored, Red contours (20% contribution) indicate regions where negatively charged substituents are favored.

The steric contour map for the CoMFA model with the most active inhibitor compound **7** is shown in Figure 4a, in which the green contours represent regions of high steric bulk tolerance (80% contribution), while the yellow contours represent regions of low steric bulk tolerance (20% contribution). It can be seen that a large green contour near C-6 of the ring B indicates a bulky group in this position is favorable to bioactivity. This is supported by the higher activity of compounds **6**–**11**, which have large substituents in that position, compared with the lower activity of compounds **4**–**5**, which have small substituents in that position. It also can be observed that there are two large yellow contours: one is next to C-3 of the ring A, and another is near C-15 and C-16 of the ring D, which suggests that a bulky group in the two regions will decrease inhibitory activity. It is confirmed by the fact that compound **17** with bulky substitution on C-3 of the ring A has lower bioactivity than compound **14** with small substitution in that position, and all the compounds with bulky groups near C-15 and C-16 of the ring D (compounds **1**–**3**, compounds **29**–**66**) show low bioactivities (pIC_50_ < 6).

The CoMFA electrostatic contour map is shown in Figure 4b, in which the red areas are the regions where a negative potential is favorable to activity, while a negative potential is unfavorable in the blue areas. This figure displays two red contours around C-3 of the ring A and one red contour near C-4 of the ring A, which means that the bioactivity can be enhanced if an electronegative atom is present in these areas. It is supported that most of the studied compounds have electronegative atoms on C-3 or C-4 of the ring A. In addition, it also can be confirmed by the fact that compound **22**, **24** with oxygen atom between C-3 and C-4 show higher activity than compound **21**, **23** with no oxygen atom in that position, respectively.

### 2.4. CoMSIA Contour Maps

**Figure 5:**
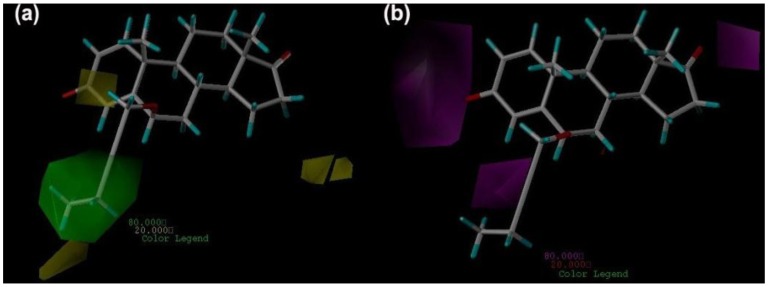
CoMSIA contour maps (standard deviation × coefficient ) in combination with compound **7**. (**a**) Steric contour maps: Green contours refer to sterically favored regions, yellow contours indicate sterically disfavored regions; and (**b**) HBA contour maps: Magenta contours show regions where HBA substituents are expected; Red contours refer to areas where HBA substituents are unexpected.

The steric and HBA fields are shown in Figure 5. For each field, the favorable and disfavored contours represent 80% and 20% level contributions, respectively. Figure 5a shows the steric contour map for the CoMSIA model with the most active inhibitor compound **7**, in which green regions are sterically favorable and yellow regions are sterically unfavorable. It is clear that the distribution of steric field in CoMSIA is basically consistent with CoMFA results. A little difference is that there is a yellow contour below the large green contour, which indicates a proper bulky substituent on C-6 of the ring B, is favorable to bioactivity while too large substituent in that position is unfavorable. This is supported by the case that the inhibitory activities of compounds **7**–**10** decline with the augment of the substituents on C-6 of the ring B.

Figure 5b shows the HBA contour maps, in which magenta and red contours represent areas where HBA substituents are favored and disfavored, respectively. There are there magenta contours in this figure: one is next to C-3 of the ring A, another is near C-4 of the ring A, and the third one is close to C-17 of the ring D, which indicates that the activity can be enhanced if the HBA atoms are present in these there positions. This is also consistent with the distribution of electrostatic field in CoMFA. It is supported by the case that all the studied compounds have at least one HBA atom in those positions. It also can be confirmed by the example that compound **13** (Formestane) show quite high bioactivity because of its three HBA atoms in those positions.

### 2.5. Pharmacophore Generation

**Figure 6:**
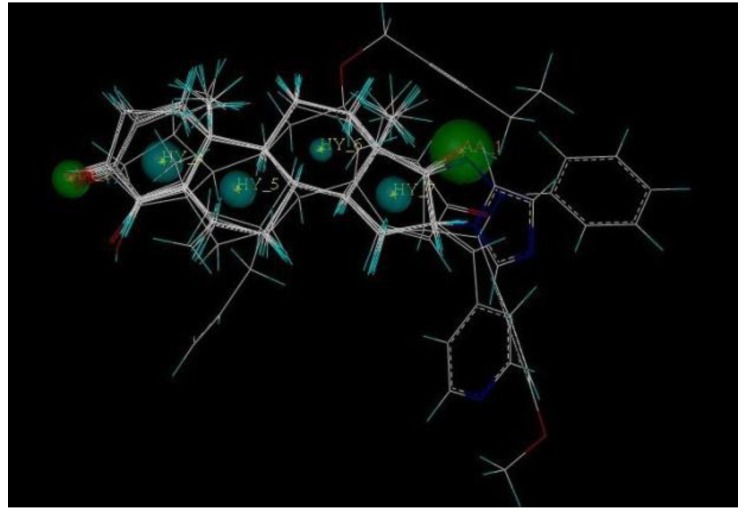
The selected GALAHAD model includes two acceptor atoms (green) and four hydrophobic centers (cyan). The sphere sizes indicate query tolerances.

Twenty pharmacophore models were generated with default parameters after Genetic Algorithm with Linear Assignment of Hypermolecular Alignment of Database (GALAHAD) run, and their statistical values are listed in Table 4. Each of the obtained models represents a different tradeoff among the conflicting demands of maximizing steric consensus, maximizing pharmacophore consensus, and minimizing energy. All the twenty models had Pareto rank 0, which means no one model is superior to any other one. Model_03 has very high energy, which is recognized that a high-energy value is due to steric clashes [30]. Small value of energy and high values of Specificity, N_hits, Sterics and Mol_Qry are desired for the best model [31]. Therefore, Model_04 was considered to be the best model and its statistical values are shown in Table 4. This model contains two acceptor atoms and four hydrophobic centers, which is shown in Figure 6 and will be converted into a 3D query for the further virtual screening studies. This pharmacophore model is consistent with the results of CoMFA and CoMSIA. Four hydrophobic centers mean the centers of A, B, C and D rings of steroids, and two acceptor atoms next to C-3 of the ring A and C-17 of the ring D indicate that hydrogen bond acceptor atoms in these two positions can enhance the activity.

**Table 4:** The statistical values of pharmacophore models after GALAHAD run.

No.	Specificity	N_hits	Features	Pareto rank	Energy	Sterics	H-bond	Mol_Qry
Model_01	5.09	8	6	0	54.31	1158.80	56.90	17.53
Model_02	3.67	8	6	0	20.85	1146.20	57.00	13.72
Model_03	5.09	8	6	0	12657.71	1151.00	56.90	28.55
**Model_04 ^a^**	**5.15**	**8**	**6**	**0**	**8.76**	**1142.60**	**56.00**	**41.94**
Model_05	4.04	8	6	0	7.43	1142.30	55.50	41.25
Model_06	4.04	8	6	0	5.39	1147.30	54.10	10.74
Model_07	4.04	8	6	0	7.83	1135.50	56.60	35.13
Model_08	4.04	8	6	0	8.63	1140.00	56.60	13.95
Model_09	4.04	8	6	0	11.75	1140.40	56.60	17.87
Model_10	4.04	8	6	0	7.23	1138.90	56.10	4.67
Model_11	4.04	8	6	0	16.69	1146.70	55.50	41.25
Model_12	5.09	8	6	0	24.98	1149.10	57.00	1.67
Model_13	4.04	8	6	0	12.79	1152.80	56.60	0.00
Model_14	4.04	8	6	0	29.82	1148.80	55.50	35.13
Model_15	4.04	8	6	0	38.52	1158.60	54.50	11.47
Model_16	5.15	8	6	0	7.25	1137.40	54.40	41.94
Model_17	5.09	8	6	0	49.49	1151.90	56.90	2.81
Model_18	3.69	8	6	0	7.07	1145.50	53.00	35.13
Model_19	3.90	8	5	0	28.40	1150.00	55.90	10.03
Model_20	4.04	8	6	0	9.09	1147.40	56.60	0.00

^a^ The selected model (Model_04) is indicated in boldface.

### 2.6. Virtual Screening

The obtained best GALAHAD model (Figure 6) was converted into a UNITY query, which was screened against NCI2000 database. The “flexible database search” option was implemented to perform virtual screening. Primary filters such as Lipinski’s rule of five, Van der Waals bumps, and QFIT (pharmacophoric match between query and the hit compound) were applied to reduce the dataset [32]. The screening of the pharmacophore query yielded six hit compounds that met the specific requirements. The pIC_50_ values of the six hit compounds were further predicted using the obtained CoMFA and CoMSIA models. Chemical structures and their predicted activity values of the hit compounds are listed in Table 5. Among the hit compounds, compounds NCI 77798 and NCI 79104 show high-predicted pIC_50_ values by both CoMFA and CoMSIA models (pIC_50_ > 6.5), which are expected to design potent and novel SAIs.

**Table 5:** Chemical structures and their predicted activity values of screened hit compounds.

Hit Compound	Structure	pIC_50_ (Predicted by CoMFA)	pIC_50_ (Predicted by CoMSIA)
NCI 51178	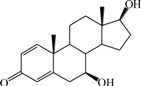	6.620	5.502
NCI 51181	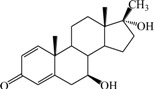	6.438	5.443
NCI 51183	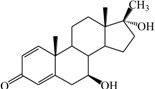	6.254	5.330
NCI 51184	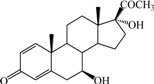	6.180	5.429
NCI 77798	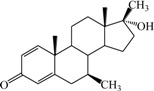	6.728	6.946
NCI 79104	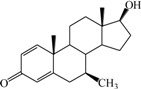	6.571	7.032

## 3. Experimental Section

### 3.1. Compounds and Biological Data

Compounds **1**–**3** [22], compounds **4**–**13** [23], compounds **14**–**17** [24], compounds **18**–**24** [25], compounds **25**–**28** [26] and compounds **29**–**66** [27] were used for this analysis, and their structures and bioactivity values are presented in Table 1. The pIC_50_ (−log IC_50_) values were used to derive 3D QSAR models. The pIC_50_ values of the studied compounds cover an interval of more than 3 log units. The whole data set of 66 compounds was divided into two groups in approximate ratio of 4:1; A training set with 50 compounds, a test set with 16 compounds (Table 1). The selection of the training and test sets was done manually such that low, moderate and high activity compounds were present in roughly equal proportions in both sets. The training set was used to build predictive models, while the test set was used to validate the predictive ability of the models.

### 3.2. Molecular Modeling and Alignment

**Figure 7:**
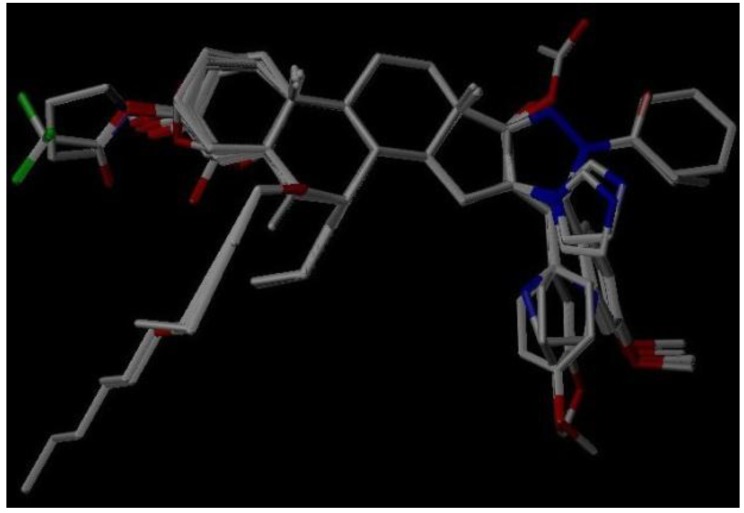
Structure of the template molecule (compound **7**), common substructure is in bold.

**Figure 8:**
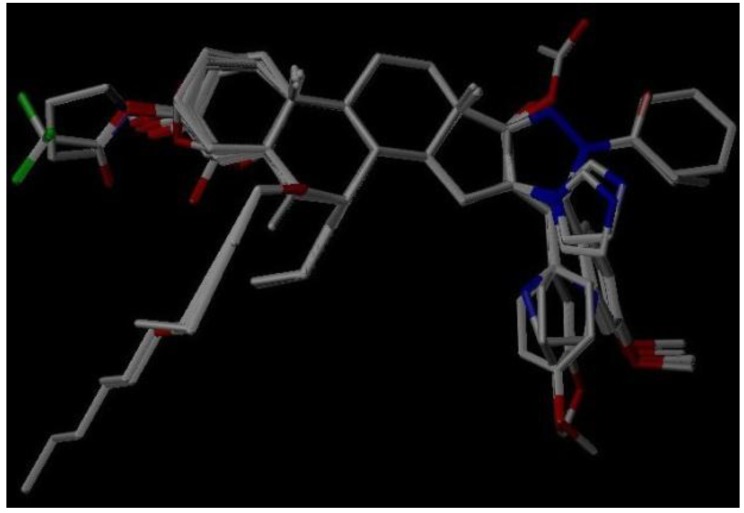
Alignment of training and test set compounds on compound **7**. Molecules are colored in white for common C, blue for N, red for O, green for F atoms, respectively.

The 3D QSAR modeling analyses, calculations and visualizations were performed using the SYBYL 7.3 molecular modeling package from Tripos Inc., St. Louis, Mo, USA, installed on Red Hat Linux workstations. Identification of the bioactive conformation is a very important step in a 3D QSAR study [33]. Among the inhibitors, the crystal structure of compound **7** bound with aromatase is available from the protein data bank (PDB code: 4GL7) [23]. Therefore, compound **7** was extracted from the complex and chosen to define the most likely binding conformation, which was modified by adding hydrogen atoms without any change of conformation and was minimized with the following steps: (i) Optimization by Steepest Descent with initial optimization of 200 simplex iterations using Tripos force field and Gasteiger-Marsili charges; (ii) Optimization by conjugate gradient; and (iii) Optimization by BFGS [34]. Three-dimensional structures of the other molecules were constructed from the compound **7**. Energy minimizations of each compound were performed according to the above procedure. In the present study, compound **7** was used as a template because of its highest activity and all other compounds were aligned on the basis of the common structure shown in Figure 7. The alignment of training and test compounds is shown in Figure 8.

### 3.3. CoMFA and CoMSIA Models

In CoMFA, the steric fields were calculated using a Lennard-Jones potential, while the electrostatic fields were calculated using a Coulombic potential. To calculate the CoMFA fields, a 3D cubic lattice with grid spacing of 2.0 Å in *X*, *Y* and *Z* directions was created automatically by SYBYL. The grid pattern extended 4.0 Å units in all directions beyond the dimensions of each molecule. The steric and electrostatic probe-ligand interaction energies were calculated by using a sp^3^ carbon probe atom and a +1.0 charge with a distance-dependent dielectric function at each lattice point. The cut-off for energies was set to ± 30 kcal/mol and the electrostatic contributions were ignored at lattice points with maximal steric interactions [17]. In CoMSIA, five different similarity fields (steric, electrostatic, hydrophobic, HBD, and HBA) were calculated. CoMSIA models were also derived with the same lattice box and all five fields were calculated using a probe of charge +1, a radius of 1, hydrophobicity and hydrogen bonding properties of +1, and an attenuation factor of 0.3 for the Gaussian distance-dependent function [18].

### 3.4. Statistical Analysis

In order to derive 3D QSAR models, CoMFA and CoMSIA descriptors were used as independent variables and the pIC_50_ values as the dependent variables. PLS method with cross-validation (leave-one-out) was used in SYBYL to determine the optimal numbers of components by using cross-validated coefficient *q*^2^ (*r*^2^_cv_). After obtaining the optimal numbers of components, a PLS analysis was performed with no validation and column filtering 2.0 to generate the final model with the training set. The obtained final non-cross-validated correlation coefficient (*r*^2^_ncv_) is a measure of the quality of the model. The predictive capability of the 3D QSAR models was determined from the predictive correlation (*r*^2^_pred_). The predicted activities for the test set were obtained from the model produced by the training set.

### 3.5. Pharmacophore Hypothesis

The pharmacophore hypothesis was generated using GALAHAD module of SYBYL, which operates in two main stages: The ligands are aligned to each other in internal coordinate space, and then the conformations produced are aligned in Cartesian space. The feature considered in developing the pharmacophore model includes HBD atoms, HBA atoms, hydrophobic and charged centers [35,36,37]. In our study, eight compounds shown in Table 1 were selected to carry out the pharmacophore hypothesis and the genetic algorithm was used to create conformers for all molecules. The compounds selected to generate the pharmacophore hypothesis are highly active and structurally diverse.

## 4. Conclusions

Aromatase inhibitors have proven to be the most important targets for treatment of estrogen-dependent cancers. In order to search for more potent SAIs with lower side effects and overcome the drug resistance, 3D QSAR studies, pharmacophore modeling and virtual screening were performed. The 3D QSAR techniques, CoMFA and CoMSIA, were applied for the first time to 66 new-synthesized SAIs, and the obtained models show good statistical results, *q*^2^ = 0.636, *r*^2^_ncv_ = 0.988, *r*^2^_pred_ = 0.658 for CoMFA and *q*^2^ = 0.843, *r*^2^_ncv_ = 0.989, *r*^2^_pred_ = 0.601 for CoMSIA, which indicate that both of the models have good quality and predictive ability. CoMFA and CoMSIA contour maps show that a proper bulky group near C-6 is favorable for activity while a bulky group near C-3, C-15 and C-16 is unfavorable, and the hydrogen bond acceptor atoms on C-3, C-4 and C-17 can enhance the bioactivity. In addition, pharmacophore models were derived from eight highly active and structurally diverse compounds using GALAHAD. The obtained best pharmacophore model includes two acceptor atoms and four hydrophobic centers, which was used as a query to search NCI2000 database. Six hit compounds were obtained after the screening of the pharmacophore query, and their pIC_50_ values were further predicted using the obtained CoMFA and CoMSIA models, which are expected to design potent and novel SAIs. The present 3D QSAR, pharmacophore modeling, and virtual screening approach provide useful information to design and synthesize potent and novel SAIs.

## Statements

As shown in a series of recent publications [38,39,40,41,42,43,44,45] in response to the call in a comprehensive review [46], user-friendly and publicly accessible web-servers represent the future direction for developing practically more useful prediction and modeling methods or demonstrating new and novel findings, we shall make efforts in our future work to provide a web-server for the approach and finding presented in this paper.
