# Correction: Xie, H.; *et al*. 3D QSAR Studies, Pharmacophore Modeling and Virtual Screening on a Series of Steroidal Aromatase Inhibitors. *Int. J. Mol. Sci.* 2014, *15*, 20927–20947

**DOI:** 10.3390/ijms16035072

**Published:** 2015-03-05

**Authors:** Huiding Xie, Kaixiong Qiu, Xiaoguang Xie

**Affiliations:** 1 Department of Chemistry, Yunnan University, Kunming 650091, Yunnan, China; 2 Department of Chemistry, School of Pharmaceutical Science and Yunnan Key Laboratory of Pharmacology for Natural Products, Kunming Medical University, Kunming 650500, Yunnan, China; E-Mail: chenneyao16@hotmail.com

A number of sentences in the first paragraph of the introduction of [28] were copied verbatim from [21,22,25,29]. Although [21,22,25] were cited in the text, [29] was omitted and it was not made sufficiently clear that direct quotations were used. The authors wish to apologize to the authors of [21,22,25,29] and to the readers of the journal for any inconvenience.

The authors wish to replace the introduction of [28] with the following:

## 1. Introduction

Aromatase is a cytochrome P-450 dependent enzyme, which catalyzes the biosynthesis of estrogens from androgens. Aromatase inhibitors (AIs) control the level of estrogens and have been effectively used in the treatments of estrogen-dependent breast cancer [1,2,3]. AIs are classified into two types: steroidal aromatase inhibitors (SAIs) and non-steroidal aromatase inhibitors (NSAIs) [4]. NSAIs bind to the enzyme active site by competing with the substrate, and they are mostly azole type compounds such as anastrozole and letrozole [5]. However, SAIs are converted by the enzyme to reactive intermediates and bind irreversibly to the enzyme active site by simulating the natural substrate androstenedione, which cause to inactivation of aromatase [6]. Among SAIs, formestane was used by intramuscular injection during the early 1990s, which is not used nowadays. Instead of formestane, exemestane is widely used because of its oral activation [7]. Though anastrazole, letrozole, and exemestane are used clinically, they still have some major side effects, such as heart problems, musculoskeletal effects, and bone toxicity [8]. For this reason, it is necessary to develop other potent and specific molecules with lower side effects.

Quantitative structure-activity relationship (QSAR) methods have been widely applied to assist the design of new drug candidates nowadays [9,10,11,12,13,14,15,16]. Comparative molecular field analysis (CoMFA) and comparative molecular similarity indices analysis (CoMSIA) are two of the most widely used three-dimensional quantitative structure-activity relationship (3D QSAR) methodologies. At various intersections of a regular three-dimensional lattice, CoMFA uses Lennard-Jones and Coulomb potential fields to calculate the energies of steric and electrostatic interactions between the compound and the probe atom, respectively. The results calculated by these two potential functions can be represented as a three-dimensional “coefficient contour” map [17]. However, in order to avoid some inherent deficiencies caused by the Lennard-Jones and Coulomb potential functions, CoMSIA calculates the energies of interactions between the molecular atoms and the probe atom by introducing Gaussian function for the distance dependence. The contour maps obtained by the CoMSIA approach can show how steric fields, electrostatic fields, hydrophobic fields, hydrogen bond donor (HBD), and hydrogen bond acceptor (HBA) influence the activity of inhibitors [18].

Pharmacophore modeling can provide valuable insight of interactions between ligands and receptors. A pharmacophore model shows the ensemble of steric and electrostatic characteristics of different compounds. Therefore, when one class of inhibitors is found, new classes of inhibitors can be discovered by a pharmacophore model, and pharmacophore searching is a good way to find various chemical structures with the same features, which is a method of choice for the first round of compound selection [19,20,21].

A series of SAIs, shown in Table 1, have been reported in the recent literatures [22,23,24,25,26,27]. To understand the structural requirements for inhibitory activity and design more potent agents, 3D QSAR studies were performed for the fist time for these SAIs using CoMFA and CoMSIA. In addition, 3D pharmacophore models were created and the selected best model was used as a 3D query for virtual screening against NCI2000 database. The biological activities of hit compounds were further predicted by using CoMFA and CoMSIA models.

## References

[1] Winer E.P., Hudis C., Burstein H.J., Wolff A.C., Pritchard K.I., Ingle J.N., Chlebowski R.T., Gelber R., Edge S.B., Gralow J. (2005). American society of clinical oncology technology assessment on the use of aromatase inhibitors as adjuvant therapy for postmenopausal women with hormone receptor-positive breast cancer: Status report 2004. J. Clin. Oncol..

[2] Perez E.A. (2006). Appraising adjuvant aromatase inhibitor therapy. Oncologist.

[3] Jordan V.C., Brodie A.M.H. (2007). Development and evolution of therapies targeted to the estrogen receptor for the treatment and prevention of breast cancer. Steroids.

[4] Brueggemeier R.W., Hackett J.C., Diaz-Cruz E.S. (2005). Aromatase inhibitors in the treatment of breast cancer. Endocr. Rev..

[5] Recanatini M., Cavalli A., Valenti P. (2002). Nonsteroidal aromatase inhibitors: Recent advances. Med. Res. Rev..

[6] Seralini G.E., Moslemi S. (2001). Aromatase inhibitors: Past, present and future. Mol. Cell Endocrinol..

[7] Hong Y., Rashid R., Chen S. (2011). Binding features of steroidal and nonsteroidal inhibitors. Steroids.

[8] Dutta U., Pant K. (2008). Aromatase inhibitors: Past, present and future in breast cancer therapy. Med. Oncol..

[9] Du Q.S., Mezey P.G., Chou K.C. (2005). Heuristic molecular lipophilicity potential (HMLP): A 2D QSAR study to LADH of molecular family pyrazole and derivatives. J. Comput. Chem..

[10] Du Q.S., Huang R.B., Wei Y.T. (2008). Multiple field three dimensional quantitative structure-activity relationship (MF-3D QSAR). J. Comput. Chem..

[11] Du Q.S., Huang R.B. (2008). Review: Recent advances in QSAR and their applications in predicting the activities of chemical molecules, peptides and proteins for drug design. Curr. Protein Pept. Sci..

[12] Du Q.S., Huang R.B., Wei Y.T. (2009). Fragment-based quantitative structure-activity relationship (FB QSAR) for fragment-based drug design. J. Comput. Chem..

[13] Prado-Prado F.J., Gonzalez-Diaz H., de la Vega O.M., Ubeira F.M., Chou K.C. (2008). Unified QSAR approach to antimicrobials. Part 3: First multi-tasking QSAR model for Input-Coded prediction, structural back-projection, and complex networks clustering of antiprotozoal compounds. Bioorg. Med. Chem..

[14] Cichero E., Fossa P. (2012). Docking-based 3D QSAR analyses of pyrazole derivatives as HIV-1 non-nucleoside reverse transcriptase inhibitors. J. Mol. Model..

[15] Zhao L.Z., Liu Y.J., Hu S.Y., Zhang H.B. (2012). 3D QSAR study of Chk1 kinase inhibitors based on docking. J. Mol. Model..

[16] Chen J., Yu R., Shen B.Z., Xu Y., Liu Y.F., Zheng H., Yao W.B. (2013). Docking-based 3D QSAR modeling of the inhibitors of IMP metallo-β-lactamase. Med. Chem. Res..

[17] Cramer R.D., Patterson D.E., Bunce J.D. (1988). Comparative molecular field analysis (CoMFA). 1. Effect of shape on binding of steroids to carrier proteins. J. Am. Chem. Soc..

[18] Klebe G., Abraham U., Mietzner T. (1994). Molecular similarity indices in a comparative analysis (CoMSIA) of drug molecules to correlate and predict their biological activity. J. Med. Chem..

[19] Sirois S., Wei D.Q., Du Q.S., Chou K.C. (2004). Virtual screening for SARS-CoV protease based on KZ7088 pharmacophore points. J. Chem. Inf. Comput. Sci..

[20] Liu L., Ma Y., Wang R.L., Xu W.R., Wang S.Q., Chou K.C. (2013). Find novel dual-agonist drugs for treating type 2 diabetes by means of cheminformatics. Drug Des. Dev. Ther..

[21] Bhatt H.G., Patel P.K. (2012). Pharmacophore modeling, virtual screening and 3D QSAR studies of 5-tetrahydroquinolinylidine aminoguanidine derivatives as sodium hydrogen exchanger inhibitors. Bioorg. Med. Chem. Lett..

[22] Bansal R., Thota S., Karkra N., Minu M., Zimmer C., Hartmann R.W. (2012). Synthesis and aromatase inhibitory activity of some new 16*E*-arylidenosteroids. Bioorg. Chem..

[23] Ghosh D., Lo J., Morton D., Valette D., Xi J., Griswold J., Hubbell S., Egbuta C., Jiang W., An J., Davies H.M. (2012). Novel aromatase inhibitors by structure-guided design. J. Med. Chem..

[24] Bansal R., Guleria S., Thota S., Bodhankar S.L., Patwardhan M.R., Zimmer C., Hartmann R.W., Harvey A.L. (2012). Design, synthesis and evaluation of novel 16-imidazolyl substituted steroidal derivatives possessing potent diversified pharmacological properties. Steroids.

[25] Valera C., Tavares da Silva E.J., Amaral C., Correia da Silva G., Baptista T., Alcaro S., Costa G., Carvalho R.A., Teixeira N.A.A., Roleira F.M.F. (2012). New structure-activity relationships of A- and D-Ring modified steroidal aromatase inhibitors: Design, synthesis, and biochemical evaluation. J. Med. Chem..

[26] Valera C.L., Amaral C., Correia-da-Silva G., Carvalho R.A., Teixeira N.A., Costa S.C., Roleira F.M.F., Tavares-da-Silva E.J. (2013). Design, synthesis and biochemical studies of new 7α-allylandrostanes as aromatase inhibitors. Steroids.

[27] Abdalla M.M., Al-Omar M.A., Bhat M.A., Amr A.E., Al-Mohizea A.M. (2012). Steroidal pyrazolines evaluated as aromatase and quinone reductase-2 inhibitors for chemoprevention of cancer. Int. J. Biol. Macromol..

[28] Xie H., Qiu K., Xie X. (2014). 3D QSAR Studies, Pharmacophore modeling and virtual screening on a series of steroidal aromatase inhibitors. Int. J. Mol. Sci..

[29] Cao H., Zhang H., Zheng X., Gao D. (2007). 3D QSAR studies on a series of potent and high selective inhibitors for three kinases of RTK family. J. Mol. Graph. Model..

